# Regulation of insulin resistance and type II diabetes by hepatitis C virus infection: A driver function of circulating miRNAs

**DOI:** 10.1111/jcmm.13553

**Published:** 2018-02-07

**Authors:** Adit Singhal, Aniruddh Agrawal, Jun Ling

**Affiliations:** ^1^ Geisinger Commonwealth School of Medicine Scranton PA USA; ^2^ Topiwala National Medical College Mumbai India

**Keywords:** bioinformatic analysis, circulating miRNA, diabetes, hepatitis C virus, insulin Resistance, microRNA, miR‐122

## Abstract

Hepatitis C virus (HCV) infection is a serious worldwide healthcare issue. Its association with various liver diseases including hepatocellular carcinoma (HCC) is well studied. However, the study on the relationship between HCV infection and the development of insulin resistance and diabetes is very limited. Current research has already elucidated some underlying mechanisms, especially on the regulation of metabolism and insulin signalling by viral proteins. More studies have emerged recently on the correlation between HCV infection‐derived miRNAs and diabetes and insulin resistance. However, no studies have been carried out to directly address if these miRNAs, especially circulating miRNAs, have causal effects on the development of insulin resistance and diabetes. Here, we proposed a new perspective that circulating miRNAs can perform regulatory functions to modulate gene expression in peripheral tissues leading to insulin resistance and diabetes, rather than just a passive factor associated with these pathological processes. The detailed rationales were elaborated through comprehensive literature review and bioinformatic analyses. miR‐122 was identified to be one of the most potential circulating miRNAs to cause insulin resistance. This result along with the idea about the driver function of circulating miRNAs will promote further investigations that eventually lead to the development of novel strategies to treat HCV infection‐associated extrahepatic comorbidities.

## 1 INTRODUCTION

The hepatitis C virus (HCV) is an enveloped, single‐stranded positive‐sense RNA virus with estimated 150‐170 million people chronically infected worldwide. Although the consequences of chronic HCV infection are generally associated with liver manifestations such as hepatic fibrosis, cirrhosis, steatosis (known as non‐alcoholic fatty liver disease, NAFLD) and HCC, the liver‐related mortality of 350 000 individuals annually is still underestimated due to the lack of consideration of extrahepatic repercussions. Among these extrahepatic complications, a growing body of evidence has particularly shown that HCV infection is strongly associated with the dysregulation of glucose homoeostasis such as insulin resistance (IR) and type 2 diabetes (T2D). Studies thus far and especially a recent retrospective study with 2435 patients^1^, ^2^, ^3^ have confirmed the relationship between HCV infection and T2D: People infected with HCV are 4 times more likely to develop T2D; clearance of HCV with direct‐acting antivirals significantly decreases HbA1c and the dependence on anti‐diabetes medications; and HCV‐infected patients with uncontrolled glucose are at higher risk to develop advanced liver fibrosis, HCC, and exhibit decreased sustained virologic response (SVR) to traditional interferon treatment.

It is estimated that 75%‐80% of HCV patients will progress to chronic infection, the stage highly associated with the development of intrahepatic complications that subsequently lead to metabolic disorders. Mechanistic studies have revealed that HCV protein NS5A and the core protein directly inhibit microsomal triglyceride transfer protein (MTP) activity, thereby reducing very low‐density lipoprotein (VLDL) assembly and inducing hepatic steatosis. In addition, HCV core protein is also associated with excessive reactive oxidative species (ROS) production via impairment of PPARγ, thereby inducing oxidative stress, mitochondrial dysfunction and steatosis. Over time, accumulation of hepatic triglycerides leads to hepatic IR via decreased insulin‐stimulated glycogen synthesis and enhanced hepatic gluconeogenesis; such conditions further cause peripheral IR in multiple organs through increased circulating insulin and free fatty acid levels.^4^, ^5^ Meta‐analyses have further confirmed the positive relationship between HCV and IR.^2^, ^6^ Furthermore, non‐diabetic and non‐obese chronic HCV patients exhibit decreased peripheral glucose uptake and hepatic IR.^7^, ^8^ Decreased glucose abnormalities have also been reported among HCV patients who have SVR after interferon treatment,^9^, ^10^ further demonstrating the role of HCV in glucose metabolism. Proposed molecular mechanisms underlying this relationship include increased serum levels of pro‐inflammatory cytokines TNF‐α, TNFR1, TNFR2, IL‐6, IL‐10, decreased adiponectin and increased intramyocellular lipocalin‐2.^8^, ^11^, ^12^, ^13^, ^14^, ^15^ These factors activate an array of intracellular signalling pathways that result in increased ROS, cellular stress and metabolic dysregulation.

There are a number of investigations on the molecular mechanisms of regulation of insulin signalling by HCV infection. Insulin‐treated liver tissue from non‐obese and non‐diabetic HCV‐infected patients results in increased levels of insulin receptor and insulin receptor substrate 1 (IRS‐1), but showing decreased levels of IRS‐1 tyrosine phosphorylation, PI3K activity and Akt phosphorylation.^16^ Similarly, HCV core protein has been found to increase serine rather than tyrosine phosphorylation of IRS‐1 in hepatocytes, resulting in its degradation and impaired downstream Akt signalling.^17^ HCV core protein also stimulates IRS‐1 serine phosphorylation via increasing mTOR levels, again resulting in decreased Akt signalling.^18^ Reduced surface expression of glucose transporters GLUT1 and GLUT2 with consequential reduction in glucose uptake in HCV‐infected hepatocytes has also been reported.^19^ Impairment of PI3K and Akt phosphorylation by HCV has also been demonstrated by several studies to be mediated via the increased level of suppressor of cytokine signalling 3 (SOCS3), which inhibits PI3K and Akt phosphorylation and stimulates IRS‐1 and IRS‐2 proteasome degradation.^20^, ^21^, ^22^ Additionally, increased ER stress response caused by HCV infection elevates protein phosphatase 2A (PP2A), also an inhibitor of Akt.^23^ Despite these findings of IR development via direct effects on insulin signalling pathway, the complex relationship between intrahepatic HCV infection and extrahepatic IR remains elusive.

Interestingly, recent evidence has implicated changes in host microRNA (miRNA) expression profiles after HCV infection. Thus, we will focus on this relationship to analyse the development of extrahepatic IR associated with HCV infection in this study. miRNAs are ~22 nucleotide (nt)‐long non‐coding RNAs that function mainly by targeting the 3′ untranslated regions (UTRs) of mRNAs, thus playing broad roles in regulating cellular functions such as development, differentiation, apoptosis, tumorigenesis, metabolism and host‐pathogen interactions.^24^ The association of specific miRNAs with certain disease states has therefore greatly accelerated the development of miRNAs into new biomarkers and/or as therapeutic targets. As miRNAs also exist in serum and other body fluids, study of extracellular and circulating miRNA profiles has become an important approach to investigate the systematic roles of miRNA in disease development. Although the functions of circulating miRNAs remain unclear, these miRNAs have been found to be highly stable under harsh conditions including boiling, pH change, long‐term storage at room temperature and multiple freeze‐thaw cycles, suggesting their important biological functions.^25^ More evidence has emerged to support the idea that circulating miRNAs can function like hormones to regulate gene expression in distant tissues and organs. The changes in circulating miRNA profiles have also been revealed to be correlated with various diseases including cancers, cardiovascular disorders and diabetes.^25^, ^26^, ^27^, ^28^, ^29^

**Figure 1:**
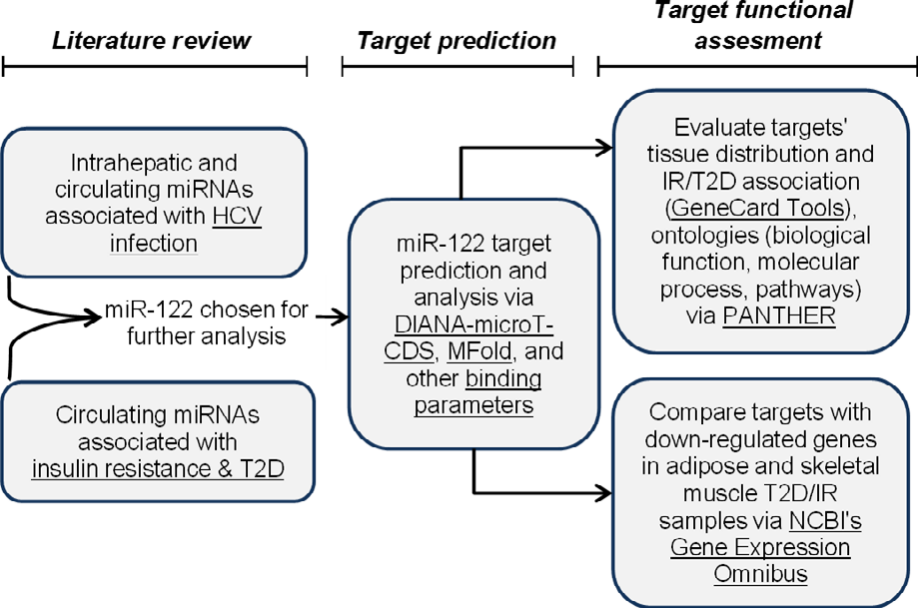
Study approach. A flow chart illustrating the methods and tools used in this study

Based on the analysis of current knowledge regarding the relationship between HCV infection and IR, we hypothesize that circulating miRNAs post‐HCV infection can target peripheral tissues and modulate their gene expression patterns, thus serving as drivers in the development of IR and T2D. Comprehensive literature review, bioinformatics and structural analyses were carried out to examine our hypothesis. The general study approach was outlined in Figure 1. We will first assess the overlap between circulating miRNAs from HCV infection and from T2D/IR, and then analyse the target genes of these miRNAs and their corresponding metabolic functions. In addition to the mechanisms described above on insulin signalling pathways, we will focus on the identification of metabolic effects associated with circulating miRNAs to improve our understanding of the complicated relationship between HCV infection and peripheral IR.

## 2 HCV INFECTION ALTERS INTRA‐ AND EXTRAHEPATIC miRNA PROFILES TO PROMOTE VIRAL PATHOGENESIS AND LIVER DISEASE

Although HCV does not directly encode miRNAs to influence cellular functions throughout the viral life cycle, changes in host miRNA expression profiles upon infection have been found to associate with various stages of HCV pathogenesis and liver diseases (Table 1). These observations have garnered attention to understanding the extracellular effects of these altered host miRNAs and their consequent functions in disease development and treatment.

**Table 1:** Hepatitis C virus (HCV) infection alters the liver miRNA profile to promote viral pathogenesis and liver disease

Hepatic miRNA	Expression	Target	Function
HCV infection			
miR‐122	Up	HCV 5′ UTR	Enhance HCV replication,^48^, ^96^, ^98^, ^99^ enhance HCV translation,^97^, ^100^ inhibit 5′ HCV RNA degradation^101^
		Xrn1	Inhibit 5′ HCV RNA degradation^102^
		Cyclin G1	Mediate alcohol‐induced increase in HCV replication^103^
		—	Reduce SOCS3 promoter methylation, inhibiting interferon‐induced ISRE activity^104^
miR‐491	Down	—	Inhibit HCV replication via down‐regulating PI3 kinase/Akt signalling^33^
miR‐130a	Up	IFITM1	Promote HCV replication via inhibiting type 1 interferon signalling^45^
miR‐21	Up	MyD88, IRAK1	Promote HCV replication via inhibiting type 1 interferon signalling^46^
miR‐27a	Up	RXRa, ABCA1	Decrease viral infectivity via regulation of lipid metabolism^52^
miR‐196	—	Bach1	Relieve Bach1 repression of antioxidant/anti‐inflammatory HMOX1, inhibit HCV translation^50^
		HCV NS5A region	Inhibit HCV replication^105^
miR‐448	—	HCV Core region	Inhibit HCV replication^105^
mir‐29	Down	—	Inhibit HCV replication^107^
miR‐199a	—	HCV 5′ UTR	Inhibit HCV replication^106^
Growth, inflammation and fibrosis			
mir‐449a	Down	—	Inhibit NOTCH signalling components and associated pro‐inflammatory marker YKL40^108^
miR‐21	Up	SMAD7	Promote fibrosis via increased TGF‐beta signalling^109^
mir‐29	Down	—	Inhibit cell proliferation and collagen expression^107^
miR‐155	Up	APC	Promote proliferation and tumorigenesis via Wnt signalling^49^
miR‐141	Up	DLC‐1	Promote cell proliferation via inhibiting tumour suppressor DLC‐1^110^
miR‐193b	Up	Mcl‐1	Promote apoptosis via inhibition of anti‐apoptotic Mcl‐1^111^
miR‐491	Down	—	Down‐regulate PI3 kinase/Akt signalling^33^
Metabolism			
miR‐27a	Up	RXRa, ABCA1	Regulation of lipid metabolism including SREB/PPAR/Apo family proteins^52^
miR‐122	Up	HCV 5′ UTR	Correlates with plasma cholesterol concentrations, miR‐122 inhibition results in decreased plasma cholesterol concentrations^53^, ^96^

ISRE, interferon‐sensitive response element. Literature review revealed several key intracellular miRNA changes upon HCV infection; their respective identified targets and associated functions are highlighted. Note that miRNAs without expression information or identified targets are indicated by —.

### 2.1 miRNA profiling

Global miRNA expression profiling highlights its importance in understanding HCV pathogenesis and developing targeted therapies. miRNA profiling of HCV‐infected human hepatoma cells revealed 108 differentially expressed miRNAs (≥2‐fold change) upon acute infection. miR‐24, miR‐149, miR‐638 and miR‐1181 were further identified to be involved in viral entry, replication and propagation, suggesting their roles in virus maintenance, survival and consequential disease.^30^ Whereas in chronic infection, virus‐promoting miR‐122 was found to be down‐regulated and antiviral miR‐296 was up‐regulated, implicating the involvement of these miRNAs and their target mRNAs in infection progression and activation of host defence responses.^31^ Similar research on miRNA profiling in HCV‐infected hepatoma cells has also identified several down‐regulated miRNAs, including miR‐30(a‐d) and miR‐130a/301, which have targets involved in viral entry, propagation and host responses. Treatment of these cells with interferon‐α restored miRNA expression, thus indicating causal roles of these miRNAs in HCV pathology.^32^ Another miRNA profiling study of HCV‐infected hepatoma cells revealed the changes of several miRNAs, including miR‐192/miR‐215 and miR‐491, which can enhance HCV replication via the suppression of PI3K/Akt pathway.^33^ miRNA profiling of HCV infection also associates with HCC development via targeting mRNAs involved in cancer‐relevant pathways such as cell cycle, adhesion, apoptosis and cell proliferation.^34^, ^35^ miRNAs identified from these studies are varied, which may be due to different samples (cell lines, fresh and archived patient specimens) and the methods used. Nevertheless, these findings clearly indicate the important roles of miRNAs in HCV pathogenesis and associated complications.

The importance of miRNAs in liver pathology is also reflected on many aspects. While serum miR‐122 levels increase at early stage of fibrosis with high inflammation, later during the progression of fibrosis to cirrhosis, both hepatic and serum levels of miR‐122 are decreased by a large cohort study with 84 liver biopsies and 167 serum samples from chronic hepatitis C patients.^36^ This is largely due to the loss of hepatocytes, which is also consistent with the inverse relationship between hepatic miR‐122 and liver injuries under various conditions.^37^, ^38^, ^39^ miR‐122 is the most abundant miRNA in liver.^40^ It is also a good biomarker for liver damage correlated with alanine aminotransferase (ALT) and aspartate transaminase (AST) activities in HCV mono‐infection and HIV/HCV con‐infection conditions.^41^, ^42^ In addition, the increase in circulating miR‐122 is greater in HIV/HCV co‐infection than HIV mono‐infection^43^; miR‐122 is also correlated with liver injury and necroinflammation (eg IL‐6) caused by HIV and HIV/HCV infections.^42^, ^44^ Based on these important findings of miR‐122 in viral infection‐induced liver diseases, we will focus on miR‐122 in this study to discuss its role in linking HCV infection with extrahepatic comorbidities.

### 2.2 Immune response

Specific changes of miRNAs with infection have been found to impact host immune response. miR‐130a expression was increased in infected hepatocytes, and liver biopsy identified a target as the antiviral protein interferon‐induced transmembrane protein 1 (IFITM1); subsequent exposure to anti‐miR‐130a increased IFITM1 expression.^45^ HCV infection has also been reported to activate miR‐21, thereby suppressing HCV‐triggered type I IFN production and promoting HCV replication. Two identified targets for miR‐21, myeloid differentiation factor 88 (MyD88) and interleukin‐1 receptor‐associated kinase 1 (IRAK1), are involved in toll‐like receptor signalling and HCV‐induced type 1 IFN production. These findings demonstrate the potential role of miR‐21 in HCV proliferation and immune surveillance evasion.^46^ Furthermore, elevated cellular miR‐122 has also been described as an essential component of HCV replication and RNA accumulation in liver cells,^47^ thus serving as an ideal candidate for antiviral treatment. Concomitantly, a recent study showed that the sequestration of miR‐122 function via subcutaneous locked nucleic acid‐modified DNA antisense oligonucleotide (drug name: Miravirsen) therapy in infected patients generated prolonged dose‐dependent reduction of HCV RNA levels without viral resistance.^48^

Intrahepatic miRNA changes from HCV infection have also been found to stimulate inflammatory signalling pathways. Induction of miR‐155 upon HCV infection via nuclear factor kappa B (NF‐κB) was revealed to inhibit apoptosis and promote hepatocyte proliferation and tumorigenesis through targeting adenomatous polyposis coli (APC), a Wnt signalling inhibitor. Wnt pathway overexpression reversed this process and induced cell cycle arrest.^49^ miR‐196 was found to inhibit HCV replication through targeting Bach1, a transcriptional factor that negatively regulates heme oxygenase 1 (HMOX1), a key cytoprotective enzyme with antioxidant and anti‐inflammatory properties involved in oxidative stress and liver injury. Overexpression of miR‐196 was therefore proposed as a potential therapeutic strategy to protect against oxidative stress and HCV infection.^50^ A similar mechanism was identified in hepatitis B virus infection, wherein miR‐143 induced hepatocarcinoma metastasis via NF‐κB signalling and fibronectin repression, thus the down‐regulation of miR‐143 became an effective strategy to control HBV‐related liver cancer progression.^51^

### 2.3 Metabolic effects

Regulation of metabolism is another key function of miRNAs from HCV infection. miR‐27a was found to regulate lipid metabolism via targeting transcription factor RXRα and the lipid transporter ATP‐binding cassette subfamily A member 1. miR‐27a also repressed several lipid metabolism‐related genes (encoding SREB, PPAR and Apo family proteins) which are essential for the production of infectious viral particles. Repression of miR‐27a was conversely found to increase hepatocyte lipid content and viral replication and infectivity. Interestingly, miR‐27a has been found to be preferentially up‐regulated during HCV infection, providing a survival mechanism by which the virus can maintain a low viral load, evade host immune surveillance, establish chronic infection and secondarily promote hepatic steatosis.^52^ A similar study on miR‐122 also demonstrated the importance of metabolic effects of miRNAs. Depletion of hepatocyte miR‐122 by locked nucleic acid anti‐miR‐122 in non‐human primates generated a dose‐dependent decrease in plasma cholesterol.^53^ These findings along with other identified miRNAs can all target insulin signalling (eg PI3K/Akt), further demonstrating a critical role of miRNAs in associating HCV infection with the development of systemic extrahepatic complications via metabolic effects.

### 2.4 Circulating miRNAs derived from HCV infection

In addition to intracellular miRNAs, extracellular circulating miRNAs have been associated with the development and progression of various diseases (eg cancer and cardiovascular disease). To better understand the linkage between HCV infection and peripheral IR and diabetes, we seek to analyse miRNAs and their target mRNAs in these 2 disease conditions. Circulating miRNA profiling of newly diagnosed T2D patients and T2D‐susceptible individuals with normal glucose tolerance consistently finds miR‐34a to be significantly up‐regulated.^54^ A separate study reports miR‐134 to be down‐regulated in omental adipocyte samples from T2D patients,^55^ and miRNA profiling of streptozotocin‐induced diabetic mice reveals miR‐134 to be up‐regulated specifically in mitochondria of liver samples.^56^ Interestingly, a few of these miRNAs in T2D/IR have also been detected in circulating miRNA profiles of HCV‐infected individuals (Table 2). A recent study on circulating miRNA profiling in HCV patient serum samples identified miR‐134, miR‐198, miR‐320c and miR‐483‐5p to be up‐regulated when compared to healthy controls.^57^ Another study by Cermelli et al^58^ found serum levels of miR‐34a and miR‐122 to be significantly elevated in HCV patients and increased with the course of infection. The overlap of miRNA profiles between these disease conditions further suggests causative roles for HCV infection‐associated miRNAs in the development of extrahepatic diabetes and IR.

**Table 2:** Circulating miRNA profiles (serum and plasma) associated with hepatitis C virus (HCV) infection and T2D/IR

Circulating miRNA	Expression with HCV infection	Expression in T2D/IR
miR‐16	Up^58^	Increased in serum T2D‐associated microvascular disease^128^
miR‐19a	Up^124^	
miR‐20a	Up^121^	
miR‐22	No change^58^	
miR‐34a	Up^58^, ^124^	Increased in serum T2D^54^; Increased in plasma T2D^127^
miR‐92a	Up^121^	
miR‐122	Up^58^, ^112^, ^113^, ^114^, ^115^, ^116^, ^117^, ^118^, ^119^, ^120^	Increased in serum of obese young adults, independently associated with IR^133^, ^134^; Increased in serum, strongly associated with development of metabolic syndrome and T2D^135^
miR‐130a	Up^124^	
miR‐134	Up^57^, ^119^	
miR‐146a	Down^124^	Increased in serum T2D^55^; increased in plasma of newly diagnosed T2D, correlates with elevated heme oxygenase‐1^132^; decreased in serum T2D and pre‐DM^125^; Decreased in serum obese/dyslipidaemic T2D vs non‐T2D obese/dyslipidaemic controls, anti‐inflammatory with increased IL‐8 and HGF^131^
miR‐155	Up^117^	Increased in exosomes of obese mice, cause IR when administered to lean mice^95^
miR‐181a	Up^123^	Increased in serum T2D, targets SIRT1^130^
miR‐192	Up^118^, ^125^	Decreased in serum T2D and pre‐DM^125^; decreased in plasma T2D, metformin increased miRNA vs placebo^126^
miR‐195	Up^125^	Decreased in plasma T2D^126^
miR‐198	Up^58^	
miR‐221	Up^122^	Increased in serum T2D^128^; increased in serum T2D, BMI/HbA1c/TG positively correlated with miRNA^129^
miR‐296	Up^124^	
miR‐320C	Up^57^	
miR‐424‐3p	Up^119^	
miR‐483‐5p	Up^57^	
miR‐494	Down^120^	
miR‐629‐5p	Up^119^	
miR‐885‐5p	Up^120^	

miRNAs changed upon HCV infection were summarized; their alterations in T2D or IR were also listed for the comparative analysis.

## 3 miR‐122 AS A UNIQUE miRNA THAT LINKS HCV INFECTION TO IR AND DIABETES

Among all HCV‐associated intra‐ and extrahepatic miRNAs, miR‐122 is the most extensively studied. Many investigations have shown its involvement and necessity in the virulence of the HCV virus, including the enhancement of HCV replication and HCV mRNA translation, inhibition of HCV 5′‐RNA degradation, mediation of alcohol‐induced HCV replication and reduction in interferon‐induced ISRE (interferon‐sensitive response element) activity via inhibiting SOCS3 promoter methylation (Table 1).

Increased circulating miR‐122 is observed in serum and plasma with HCV infection, and circulating miRNA‐122 is elevated in serum of obese young adults and patients with T2D and IR (Table 2). These lines of evidence prompt us to carry out in‐depth analysis on the central roles of miR‐122 in the development of IR and T2D under HCV infection. The related peripheral targets of miR‐122 and their functions in metabolic and signalling pathways are comprehensively examined as follows.

### 3.1 miR‐122 target profiling on metabolic ontologies in peripheral tissues

**Figure 2:**
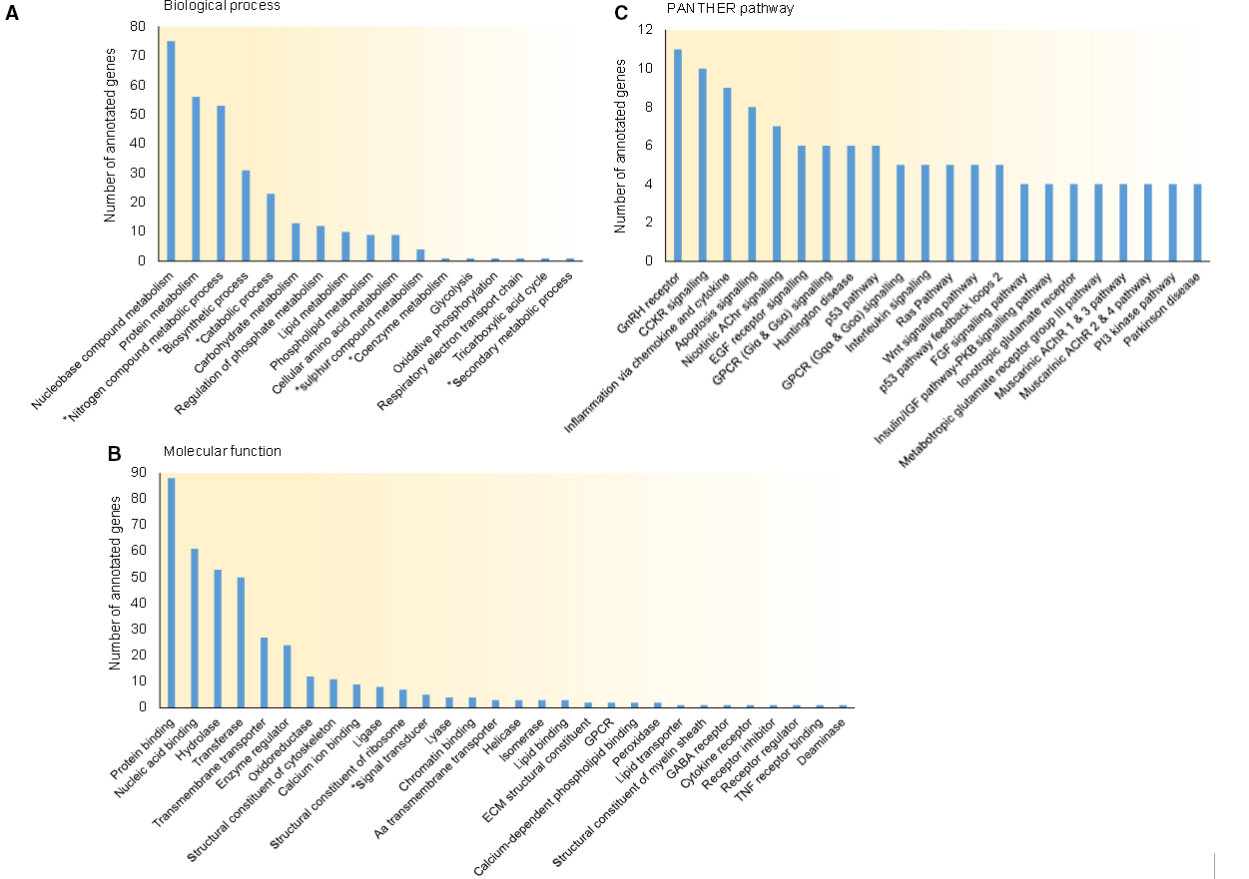
miR‐122 target profile reveals IR‐relevant ontologies and pathways. miR‐122 predicted targets were analysed for molecular function, biological process and associated pathways with PANTHER Gene Ontology Tool. (A) Biological process ontology includes protein metabolism (56), carbohydrate metabolism (13) and lipid metabolism (10). (B) Molecular function ontology revealed several IR‐relevant biochemical processes including transmembrane transport (27), calcium ion binding (9), GPCR activity (2), peroxidase activity (2) and lipid transporter activity (1). (C) PANTHER pathway analysis identified key signalling pathways including apoptosis signalling (8), EGFR signalling (6), GPCR signalling (11), Ras pathway (5), insulin/IGF pathway‐PKB signalling (4) and PI3 kinase pathway (4). *Indicates a general level 1 ontology in (B) and level 2 ontology in (A), all other listed ontologies are more specific terms at either level 2 in (B) or level 3 in (A). DIANA‐microT‐CDS predictions for miR‐122 are provided in Data S1. PANTHER results with ontologies and corresponding gene lists are provided in Data S2

To understand the potential metabolic effects of circulating miR‐122 in peripheral tissues, we first analysed the biological and molecular functional landscape of all miR‐122 targets. Using the DIANA‐microT‐CDS target prediction platform,^59^, ^60^ we identified a total 511 strongly predicted targets for miR‐122; these targets were further assessed by the PANTHER Gene Ontology database^61^ for the clarification of their molecular functions and involvement in biological processes and signalling pathways (Figure 2). As shown in Figure 2A, many metabolic processes are potentially targeted by miR‐122, including protein metabolism, carbohydrate metabolism (glycolysis, oxidative phosphorylation and tricarboxylic acid cycle), lipid metabolism and phospholipid metabolism. Many biological functions are also affected, including transmembrane transport, calcium‐dependent phospholipid binding, lipid binding and transport, and peroxidase activity etc. (Figure 2B). Signalling pathway ontology revealed several IR‐related pathways (eg insulin/IGF/AKT signalling), PI3K signalling, apoptosis, EGF receptor signalling, GPCR signalling and Ras/MAPK pathway (Figure 2C). Interestingly, inflammation ontology is also affected by miR‐122 targets, including chemokine and cytokine signalling (eg TNF receptor), interleukin signalling, Wnt signalling and cytokines.

**Figure 3:**
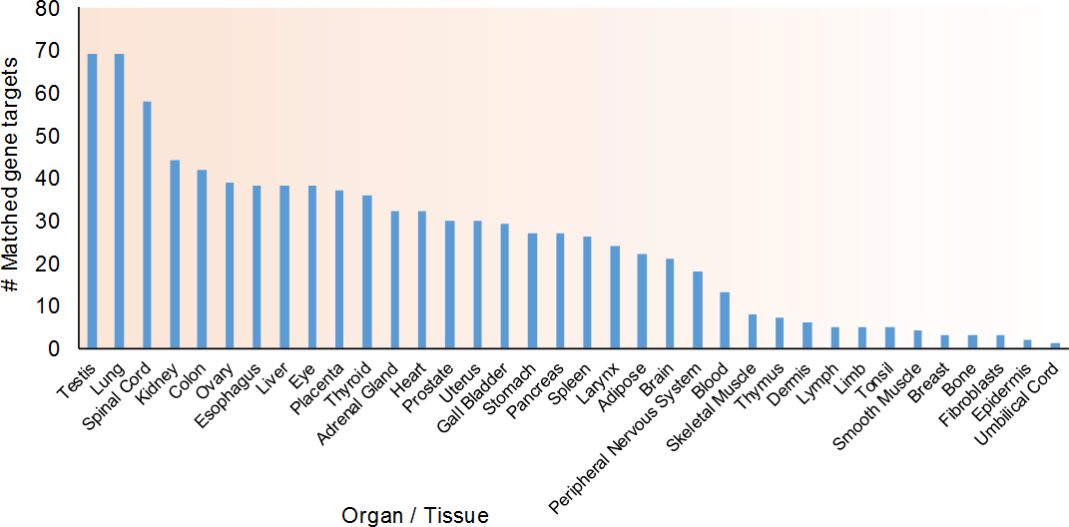
miR‐122 targets IR‐relevant genes differentially expressed in adipose, skeletal muscle and pancreas. Distribution of miR‐122 predicted targets (511 input genes) differentially expressed in various tissues and compartments was analysed via GeneAnalytics LifeMap Tool, including adipose (22), skeletal muscle (8) and pancreas (27)

Furthermore, we used GeneAnalytics LifeMap software, which analyses an input gene set against background genes known to be differentially expressed in all tissues, to analyse which targets are differentially expressed in which peripheral tissues.^62^ As shown in Figure 3, several targets were identified to be more expressed in the liver as well in several extrahepatic tissues including thyroid, pancreas, adipose, skeletal muscle and smooth muscle. Several genes differentially expressed in adipose, skeletal muscle and pancreatic tissues with metabolic functions are experimentally verified targets of miR‐122 (Table 3), further suggesting the strong possibility that miR‐122 plays critical roles in the development of IR and T2D.

**Table 3:** miR‐122 targets differentially expressed in adipose, skeletal muscle and pancreas

Target	Name	Function
Adipose		
CS	Citrate synthase	TCA cycle; conversion of glucose to acetyl‐CoA
IGFBP5	Insulin‐like growth factor‐binding protein 5	Regulation of cell growth and metabolism by IGF
ACSS2	Acyl‐CoA synthetase short‐chain family member 2	Activation of acetate for use in lipid synthesis and energy generation; ethanol oxidation
IQGAP1^a^	IQ motif containing GTPase‐activating protein 1	Insulin signalling via PKB/Akt pathway; MAPK/ERK signalling cascade
ALDOA^a^	Aldolase, Fructose‐Bisphosphate A	Glycolysis and gluconeogenesis
CLIC4^a^	Chloride intracellular channel 4	Chloride channel insertion, redox regulated, possibly under oxidizing conditions
SUMF2	Sulfatase‐modifying factor 2	Sphingolipid metabolism
Skeletal muscle		
GYS1^a^	Glycogen synthase 1	Glucose metabolism; insulin‐induced glycogenesis
NOS1	Nitric oxide synthase 1	Nitric oxide synthesis from l‐arginine
SOX6^a^	SRY‐Box 6	Transcription activator involved in Wnt and ERK signalling
ATP1A2^a^	ATPase Na^+^/K^+^ transporting unit alpha 2	Membrane electrochemical gradient regulation; muscle electrical excitability
Pancreas		
CANX^a^	Calnexin	Ca^2+^ binding, ER‐associated chaperone; Glycoprotein folding
KIF11^a^	Kinesin Family Member 11	Kinesin; microtubule‐associated secretory protein transport
ATP1A2^a^	ATPase Na^+^/K^+^ transporting unit alpha 2	Membrane electrochemical regulation; Inhibited in Ca^2+^ influx and insulin secretion
GPX3	Glutathione peroxidase 3	Secreted; Antioxidant (H2O2, lipid peroxides, organic hydroperoxides reduction by glutathione)

Targets that are differentially expressed (per GeneAnalytics LifeMap tool) and involved in IR‐relevant functions are listed.

a Experimentally verified targets in DIANA‐microT‐CDS (targets verified by microarray, immunoprecipitation or qRT‐PCR).

In adipose tissue, a number of genes with relevant metabolic functions were identified, including conversion of glucose to acetyle‐CoA (*CS*), regulation of cell growth and metabolism by IGF (*IGFBP5*), insulin signalling via PI3K/Akt and MAPK/ERK signalling (*IQGAP1*), glycolysis and gluconeogenesis (*ALDOA*), and sphingolipid metabolism (*SUMF2*). In skeletal muscle, additional genes with IR‐relevant functions were also identified, including glucose metabolism and insulin‐induced glycogenesis (*GYS1*), Wnt and ERK signalling (*SOX6*), and nitric oxide synthesis (*NOS1*). Similarly, genes relevant to insulin secretion in pancreatic tissue were identified to include calcium binding and glycoprotein folding (*CANX*), microtubule‐associated secretory transport via kinesins (*KIF11*) and membrane electrochemical regulation (*ATP1A2*).

### 3.2 miR‐122 target genes regulate IR and T2D in skeletal muscle and adipose tissue

To further evaluate miR‐122 targets that play important roles in clinical IR tissues, we analysed the relationship between miR‐122 targets and the down‐regulated genes (≥2‐fold decrease) in human IR tissues using the microarray data from 4 studies in Gene Expression Omnibus (GEO)^63^; 2 assessing skeletal muscle and 2 assessing adipose tissue (Table 4). Several miR‐122‐predicted targets are down‐regulated genes in quadriceps muscle reported by Jin et al (normoglycaemic IR subjects with parental history of T2D vs normoglycaemic non‐IR control) and Yang et al (insulin sensitive and IR from equally obese, non‐diabetic Pima Indians).^64^, ^65^ IR‐relevant genes include NaCl absorption (*SLC12A1*), insulin receptor kinase phosphorylation (*PTPN1*), IGFBP4 and IGFBP5 cleavage (*PAPPA*), and TGF‐β signalling (*PEG10*). To further examine the extent of functional association of these genes with IR, we used the GeneAnalytics VarElect software (an integrated knowledge‐based software to analyse direct and indirect phenotypic linkage with genes) and identified several miR‐122 targets (*SLC12A1, PTPN1 and PAPPA*) that have direct phenotypic association with T2D/IR.^66^ Comparing miR‐122 targets with down‐regulated genes in subcutaneous and omental adipose samples^67^ (insulin sensitive vs IR obese subjects) and subcutaneous adipose samples^68^ (insulin sensitive vs IR non‐obese subjects), we again found overlapping genes with IR‐relevant functions, including stimulation of oestrogen and glucocorticoid receptors, fat oxidation and non‐oxidative glucose metabolism (*PPARGC1B*), GPCR signalling and carbohydrate metabolism (*GRK3*), adipocyte differentiation and apoptosis inhibition (*PEG10*), stimulation of Wnt signalling (*RSPO1*), O‐linked oligosaccharide synthesis (*GALNT12*) and MAPK signalling (*DUSP4*). *PPARGC1B*,* GPD1L* and *RSPO1* were found to have more direct phenotypic association with T2D/IR when assessed by VarElect. Several of the miR‐122 targets highlighted in Table 4 in both skeletal muscle and adipose tissue have been previously experimentally verified as well.

**Table 4:** miR‐122 targets genes that are down‐regulated in IR skeletal muscle and adipose tissue

Target	Name	Function	VarElect	
			Direct	Indirect
Skeletal muscle (Quadricep, normoglycaemic IR with parental T2D history, Jin et al)				
SLC12A1	Solute carrier family 12 member 1	Na/Cl absorption; ionic balance, cell volume	●	
PTPN1^a^	Protein tyrosine phosphatase, non‐receptor type 1	Negative regulator of insulin signalling via dephosphorylating the phosphotyrosine residues of insulin receptor kinase	●	
ZNF101	Zinc finger protein 101	Transcription regulation		●
TTL	Tubulin tyrosine ligase	Post‐translational modification of alpha‐tubulin; ligase		
MYB^a^	MYB proto‐oncogene, transcription factor	Transcriptional activator		
Skeletal muscle (Quadricep, IR non‐diabetic Pima Indians, Yang et al)				
PAPPA^a^	Pappalysin 1	Metalloproteinase which specifically cleaves IGFBP‐4 and IGFBP‐5, resulting in release of bound IGF	●	
PEG10	Paternally expressed 10	Prevents apoptosis in hepatocellular carcinoma; inhibits the TGF‐beta signalling by interacting with the TGF‐beta receptor ALK1		●
CDH6	Cadherin 6	Calcium‐dependent cell adhesion proteins; Wnt signalling		●
MYO9A	Myosin IXA	Actin‐based motor molecules with ATPase activity		●
ENY2	ENY2, transcription and export complex 2 subunit	mRNA export coupled transcription activation		
Adipose (Subcutaneous, IR obese, Hardy et al)				
PPARGC1B	PPARG coactivator 1 beta	Stimulates activity of several transcription factors and nuclear receptors, including oestrogen receptor alpha, nuclear respiratory factor 1 and glucocorticoid receptor; may be involved in fat oxidation, non‐oxidative glucose metabolism and the regulation of energy expenditure; down‐regulated in pre‐diabetic and T2D patients	●	
RPS6KC1	Ribosomal protein S6 kinase C1	Sphingosine‐1 phosphate (SPP)‐mediated signalling		●
GRK3	G‐protein‐coupled receptor kinase 3	Phosphorylates the agonist‐occupied form of the beta‐adrenergic and related GPCRs		●
PEG10	Paternally expressed 10	Prevents apoptosis in hepatocellular carcinoma; adipocyte differentiation; inhibits the TGF‐beta signalling by interacting with the TGF‐beta receptor ALK1		●
Adipose (Omental, IR obese, Hardy et al)				
GPD1L	Glycerol‐3‐phosphate dehydrogenase 1‐like	Catalyses conversion of sn‐glycerol 3‐phosphate to glycerone phosphate	●	
RSPO1	R‐spondin 1	Ligand for leucine‐rich repeat‐containing G‐protein‐coupled receptors (LGR proteins) and positively regulates the Wnt signalling pathway	●	
MYO9A	Myosin IXA	Actin‐based motor molecules with ATPase activity		●
TMTC2	Transmembrane/tetratricopeptide repeat‐containing 2	ER membrane protein; ER calcium homoeostasis		●
GALNT12	Polypeptide N‐acetylgalactosaminyltransferase 12	Catalyses initial step in O‐linked oligosaccharide biosynthesis		
GRK3	G‐protein‐coupled receptor kinase 3	Phosphorylates the agonist‐occupied form of the beta‐adrenergic and related GPCRs; carbohydrate metabolism		
Adipose (Subcutaneous, IR non‐obese, Keller et al)				
DUSP4^a^	Dual specificity phosphatase 4	Negatively regulates members of the mitogen‐activated protein (MAP) kinase superfamily (MAPK/ERK, SAPK/JNK, p38), which are associated with cellular proliferation and differentiation		

Predicted miR‐122 targets were compared with down‐regulated genes (≥2 fold, *P* < .05) in skeletal muscle and adipose tissue samples in 4 reported microarray studies using Gene Expression Omnibus (GEO). Targets that are more likely associated (direct or indirect) with IR‐relevant phenotypes were determined using VarElect (phenotype query: insulin resistance, glucose intolerance, lipid metabolism, type 2 diabetes, glucose metabolism, lipid storage and inflammation).

a Experimentally verified targets (DIANA‐microT‐CDS). Lists of all overlapped genes from above 5 comparisons are available in Data S3.

## 4 MOLECULAR MECHANISMS TO SUPPORT THE FUNCTION OF CIRCULATING miR‐122 IN TARGET CELLS

Whether circulating miRNAs are functionally significant or simply by‐products of cell activity is still debatable; however, strong evidence for both sides of this relationship has emerged.^69^ Circulating miRNAs can be co‐purified with microvesicles and exosomes,^70^, ^71^ miRNA populations in exosomes are significantly different from miRNA species in their originating cells and are often associated with AGO proteins,^72^, ^73^, ^74^ and the treatment of cells with exosome fractions suppresses target mRNA translation.^28^, ^75^, ^76^, ^77^, ^78^, ^79^ All of these results support the “cell‐to‐cell communication” theory for circulating miRNAs. Conversely, opponents have shown that miRNA‐AGO complexes are stable for prolonged periods of time in the nuclease‐rich extracellular environment after the death of parental cells^80^ and suggest that miRNA‐AGO complex with exosomes is simply due to the high capacity of RNA‐binding proteins^81^, ^82^ and that the concentration of miRNAs in circulation is too low to carry out a hormone‐like function on distant target cells. Both sides of this argument may be correct to a certain extent. While the majority of circulating miRNAs might be non‐specific by‐products, certain miRNAs may indeed possess hormone‐like functions under certain conditions (eg cancer and infection).

Interestingly, vesicle‐associated miRNAs are found to be the minimal portion in circulation, whereas over 90% of miRNAs in serum and plasma are vesicle free and associated with AGO2.^76^ As AGO2 is a critical intracellular protein in the RNA‐induced silencing complex (RISC), this finding indicates a drastic new possibility that AGO2 not only functions as a carrier protein for circulating miRNAs but also plays an active role in miRNA uptake and functioning in target cells. Although the mechanism underlying the secretion, circulation and uptake of AGO2‐miRNA complex remains elusive, these findings prompt us to analyse the possibility of AGO2‐mediated uptake and functioning of miR‐122 in target cells.

### 4.1 Thermodynamic stability of miR‐122/AGO2 complex

Among 4 AGO proteins (AGO1‐AGO4) encoded by the human genome, only AGO2 expresses mRNA cleavage activity while other AGOs (AGO1/3/4) lack cleaving activity and only mediate RNA silencing via translational repression.^83^ The 2 steps of forming active RNA‐induced silencing complex (RISC) involves pre‐miRNA duplex association with AGO (inactive RISC) followed by the rate‐limiting step of RNA duplex cleavage to form a single‐stranded RNA associated with AGO (active RISC).^84^, ^85^

**Figure 4:**
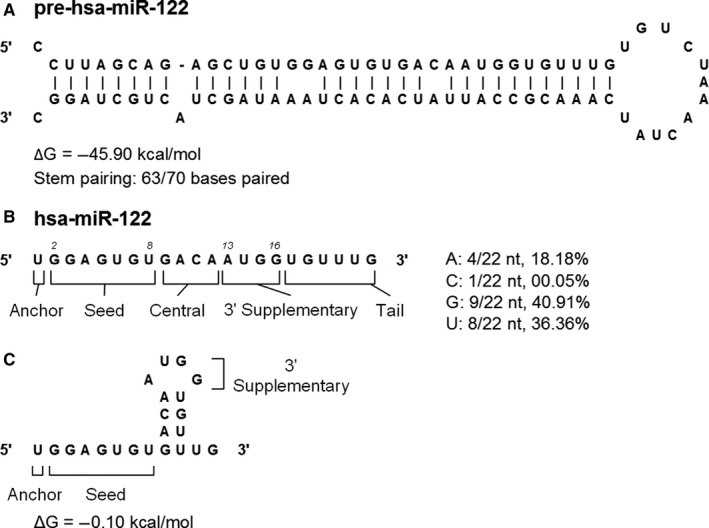
miR‐122 structural and thermodynamic characteristics make it an ideal candidate for AGO2‐mediated silencing. miR‐122 sequence was folded with the mFold RNA tool to assess its stem‐loop structure features and thermodynamic stability. (A) pre‐miR‐122 stem‐loop structure and thermodynamic stability. (B) miR‐122 sequence, relevant segments and nucleotide composition. (C) miR‐122 guide strand stem‐loop structure and thermodynamic stability. “hsa‐” stands for homo sapiens

Thermodynamic stability and structure prediction for pre‐miR‐122 by mFold revealed the ΔG = −45.90 kcal/mol along with 63/70 bases paired in its stem (Figure 4A).^86^ Gu et al demonstrated that stable shRNA structures (ΔG < −38.5 kcal/mol) are inversely correlated with non‐cleaving AGO‐RISC activation, with very poor to no activation around ‐45.90 kcal/mol. It was also demonstrated that AGO2‐associated shRNAs in complete or near complete base pairing in the stem exhibits very robust mRNA target silencing.^85^ Together, miR‐122 is likely a poor candidate for AGO1/3/4‐RISC activation, hence most likely will function via AGO2‐mediated silencing. Due to this high thermodynamic stability, we believe that miR‐122 is specifically evolved for RNA silencing function through AGO2.

Although current understanding of miRNA guide strand secondary structure formation remains limited, mFold results of miR‐122 interestingly revealed the most stable form (ΔG = −0.10 kcal/mol) to possess unpaired bases in anchor, seed, 3′ supplementary and in the later tail regions (Figure 4C). This confirmation may be ideal as it exposes only bases that are required for mRNA targeting.

Studies have also shown that miRNAs containing high percentage of adenine exhibit reduced mRNA targeting activity. It was also found that AGO2‐associated miRNAs contain less adenine nucleotides at their 3′‐end when compared to miRNAs extracted from the whole cell.^87^ Consistent with this rule, we noticed that miR‐122 contains more G (40.91%) and U (36.36%) bases than A bases (18.18%) in the regions outside of the 3′‐end, further supporting miR‐122 as a strong candidate for functioning through AGO2 (Figure 4B).

### 4.2 3′ End importance: protection from nuclease digestion

Increasing studies indicate the importance of nucleotide composition at the 3′‐end of miRNAs. Although miRNAs become more stable when bound with RISC, they are still susceptible to nuclease degradation via miRNA 3′ trimming, wherein the 3′‐end nucleotides are removed by cellular nucleases while bound to the PAZ domain of AGO proteins. This trimming process is only for AGO2‐bound miRNA but not for non‐cleaving AGO1/3/4‐associated miRNAs, suggesting a potential bias for AGO proteins towards certain nucleotide sequences.^88^, ^89^ miRNA with 3′‐end adenine nucleotides shows weak interaction with PAZ domain, whereas this interaction becomes stabilized when U/G/C nucleotides (U>G>C>A) are present at the 3′‐end.^89^, ^90^ As miR‐122's 3′‐end nucleotide is G and all 6 nucleotides in the 3′ tail are U or G, miR‐122 likely exhibits strong binding to the AGO2 PAZ domain, thus protecting it from cellular nuclease cleavage (Figure 4B).

Several studies have identified a new phenomenon for miRNA sorting: 3′‐end adenylated miRNAs are enriched in cells while 3′‐end uridylated miRNAs tend to be enriched in extracellular microvesicles.^91^ The discovery of specific miRNA 3′‐end sequence motifs (EXOmotifs) favours the loading into exosomes through the interaction with nuclear ribonucleoprotein A2B1 (hnRNPA2B1).^92^ However, miR‐122 does not meet these criteria with 3′‐end guanine and the lack of identified EXOmotifs. Therefore, other unknown mechanism(s) may be involved in the loading of miR‐122 into exosomes. As mentioned above, miR‐122 may simply form a complex with AGO2 instead of exosome packaging for efficient circulation.

### 4.3 Can AGO2 mediate miR‐122 uptake in target cells?

By the time miRNA‐AGO2 complex reaches target cells, there is another critical step for miRNA uptake. So far, there are no studies addressing this issue; it is not known whether AGO2 can mediate miRNA uptake or induce an endocytosis process. However, there is research reporting that AGO2 is localized on rough ER membrane and can bind to miRNA to cause its release from ER to target cellular mRNA.^93^ In addition, another study also identified the association of AGO2 with plasma membrane in NK cells,^94^ suggesting that AGO2 might have a potential membrane function to assist miR‐122 uptake by target cells. AGO2 might also be able to interact with other endocytosis complex protein(s) to facilitate miR‐122 uptake. This is an untested but significant topic that warrants further investigation.

## 5 CONCLUSIONS

**Figure 5:**
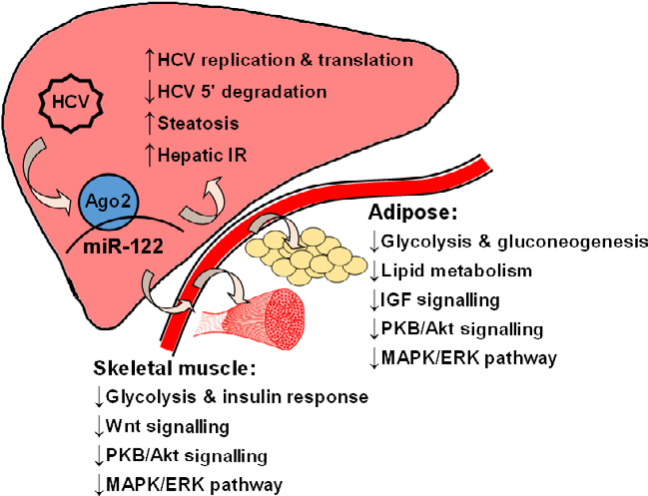
Working model for hepatic and circulating miR‐122 to mediate IR and diabetes associated with Hepatitis C virus (HCV) infection. Key metabolic and cell signalling pathways impacted are illustrated, summarizing the findings from literature review and bioinformatics analyses in this study

Through systematic literature review and comprehensive bioinformatic analysis, we identify a group of circulating miRNAs with strong potential in regulating the development of insulin resistance and diabetes‐associated HCV infection. These miRNAs are revealed by a new idea to analyse the functional overlap between HCV‐derived and IR/diabetes‐related miRNAs. By integrated analyses using DIANA‐microT, PANTHER Gene Ontology Tool, GeneAnalytics LifeMap Tool, Gene Expression Omnibus (GEO) and VarElect, miR‐122 is uncovered as the most relevant miRNA in linking HCV infection to IR/diabetes. The effects of miR‐122 on target mRNAs in insulin effector tissues are partially supported by experimental data at basic and pre‐clinical levels. The broader coverage of bioinformatic analysis than experimental research is certainly reasonable, which also provides us with more targets for further studies. The overall view of the regulatory functions of miR‐122 on the development of IR/diabetes by HCV infection is summarized in Figure 5.

The feasibility of regulating these broad mRNAs by miR‐122 is also analysed de novo by several bioinformatic methods, thus providing the biochemical and cell biology basis for the functioning of miR‐122 in extrahepatic tissues. Our viewpoint regarding the “hormonal” function of miR‐122 is supported by a recent study that obese mice adipose tissue macrophage‐derived exosome can cause systemic insulin resistance and glucose intolerance in lean mice, wherein miR‐155 is the causative factor.^95^ This study also suggests that other miRNAs identified in Table 2 may exert functions in the association of HCV infection with IR and diabetes. Interestingly, miR‐155 is exactly on the list as one candidate in Table 2. miRNAs identified from this analytical review will be experimentally verified as our ongoing research project. In summary, the information and ideas from this review shed new light on our understanding about the development of IR and diabetes associated with HCV infection from a different angle through non‐coding RNA, thus providing us a new strategy for drug development to treat HCV infection‐associated extrahepatic diseases and complications.

## CONFLICT OF INTEREST

The authors declare that they have no conflict of interest.
